# The VieB auxiliary protein negatively regulates the VieSA signal transduction system in *Vibrio cholerae*

**DOI:** 10.1186/s12866-015-0387-7

**Published:** 2015-03-04

**Authors:** Stephanie L Mitchell, Ayman M Ismail, Sophia A Kenrick, Andrew Camilli

**Affiliations:** Howard Hughes Medical Institute and the Department of Molecular Biology and Microbiology, Tufts University, School of Medicine, Boston, USA; Wyatt Technology Corporation, Santa Barbara, USA

**Keywords:** Two-component system, Phosphorelay, Bacterial signal transduction, Protein phosphorylation, Phosphodiesterases, Tetratricopeptide repeat domain, *Vibrio cholerae*

## Abstract

**Background:**

*Vibrio cholerae* is a facultative pathogen that lives in the aquatic environment and the human host. The ability of *V. cholerae* to monitor environmental changes as it transitions between these diverse environments is vital to its pathogenic lifestyle. One way *V. cholerae* senses changing external stimuli is through the three-component signal transduction system, VieSAB, which is encoded by the *vieSAB* operon. The VieSAB system plays a role in the inverse regulation of biofilm and virulence genes by controlling the concentration of the secondary messenger, cyclic-di-GMP. While the sensor kinase, VieS, and the response regulator, VieA, behave similar to typical two-component phosphorelay systems, the role of the auxiliary protein, VieB, is unclear.

**Results:**

Here we show that VieB binds to VieS and inhibits its autophosphorylation and phosphotransfer activity thus preventing phosphorylation of VieA. Additionally, we show that phosphorylation of the highly conserved Asp residue in the receiver domain of VieB regulates the inhibitory activity of VieB.

**Conclusion:**

Taken together, these data point to an inhibitory role of VieB on the VieSA phosphorelay, allowing for additional control over the signal output. Insight into the function and regulatory mechanism of the VieSAB system improves our understanding of how *V. cholerae* controls gene expression as it transitions between the aquatic environment and human host.

## Background

*Vibrio cholerae* is a human small intestinal pathogen that causes profuse secretory diarrhea and vomiting leading to severe dehydration, which if left untreated can result in death. Upon ingestion of *V. cholerae* from a contaminated water source, this pathogen travels to the small intestine where expression of the toxin coregulated pilus results in colonization and subsequent expression of virulence genes, such as cholera toxin (CT). Translocation of CT into the epithelial cells lining the small intestine leads to profuse secretory diarrhea and results in the exit of *V. cholerae* from the host back into the aquatic environment [1-4]. The lifestyle of *V. cholerae* involves two drastically different environments, the aquatic environment and the small intestine of the human host. For optimal fitness, *V. cholerae* must be able to sense these environmental changes and it therefore harbors an array of systems that link environmental stimuli to gene expression changes. Bacteria commonly use two-component systems (TCS) to sense a wide variety of environmental cues and transmit this information intracellularly [5-7]. Additionally, given that some bacteria, like *V. cholerae*, endure dramatic environmental changes as they enter the human host, a number of TCSs have been shown to play a role in bacterial pathogenesis [8-11].

*V. cholerae* has served as a model organism for studying signal transduction and regulation of virulence genes. One way *V. cholerae* senses changing environments is through the VieSAB signal transduction system, which was previously discovered through a genetic screen for virulence gene regulators [12]. VieSAB is encoded in a putative operon harboring three genes, *vieS*, *vieA*, and *vieB*. VieS is a complex sensor kinase (SK) containing two putative periplasmic ligand-binding domains and cytoplasmic histidine kinase (HK), receiver domain (REC) and histidine phosphotransferase domains (Hpt). VieS is capable of autophosphorylating and uses the complex phosphorelay system to transfer phosphate to the response regulator (RR), VieA [13]. VieA resembles a traditional RR, containing an N-terminal REC domain and C-terminal helix-turn-helix (HTH) domain. However, VieA is unusual in also harboring a cyclic-di-GMP (cdGMP) phosphodiesterase (PDE) domain that lies between the REC and HTH domains [12]. *V. cholerae* uses VieA to modulate its intracellular cdGMP concentration in order to regulate gene expression during the transition between the aquatic environment and the human host [14,15]. Lowering the concentration of cdGMP early during infection promotes expression of virulence and motility genes and leads to repression of environmental survival genes, namely biofilm formation genes [16,17]. Indeed, VieS and VieA have been implicated in the negative regulation of biofilm formation, are required for the colonization of the infant mouse, and are important for the positive regulation of the CT genes (*ctxAB)* and a major virulence gene transcriptional regulator (*toxT*), highlightening the role of VieSAB in modulating *V. cholerae* pathogenesis [13-15,18,19]. Additionally, *vieA* transcription is shown to increase significantly after the binding of *V. cholerae* to epithelial cells, supporting the hypothesis that VieA is a major contributor to cdGMP cleavage early during infection and is capable of influencing gene expression through this mechanism [20]. These studies point to a role of VieSAB in tying together the sensing of external stimuli with changes in the cytoplasmic cdGMP concentration, allowing *V. cholerae* to adapt as it transitions between the environment and the host.

However, the role of the third component in the VieSAB system, VieB, remains unclear. While VieB contains an N-terminal REC domain, it lacks the typical RR C-terminal HTH domain. Instead, it contains a tetratricopeptide repeat domain, which mediates protein-protein interactions, followed by a C-terminal half of the protein that has no known sequence homology, providing little insight into the function of this protein. Since a majority of well-studied auxiliary proteins have been described as inhibitors [21], we hypothesize that VieB acts as an inhibitor of the VieSA TCS. In this study, we uncover and characterize the function of VieB and reveal its mechanism of action.

## Results

### VieB is a dose-dependent inhibitor of VieA-His_6_ phosphorylation

**Figure 1:**
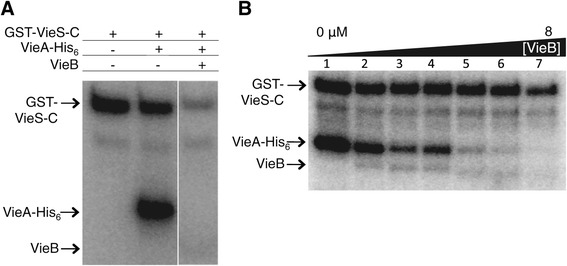
**VieB is a dose-dependent inhibitor of phosphotransfer. (A)** Purified GST-VieS-C was incubated with ^32^P-ATP-γ either alone (lane 1) or in the presence of equimolar VieA-His_6_ (lane 2) and 5 μM VieB (lane 3) for 30 minutes at 30°C. **(B)** GST-VieS-C was incubated with ^32^P-ATP-γ in the presence of equimolar VieA-His_6_ and either 0, 0.25, 0.5, 1, 2, 4, or 8 μM VieB for 30 minutes at 30°C. Samples were stopped with the addition of 2X-denaturing sample buffer and separated using a 10% SDS-Page gel. Proteins labeled with ^32^P were observed by radioautography. Figure panels are from the same experiment and exposed for the same amount of time. The radioautographs shown are a representative of three replicates. The band just below GST-VieS-C is a VieS degradation band, which is present in all figures and should be ignored.

Since many other described auxiliary proteins of TCSs negatively modulate their cognate TCS, we hypothesized that VieB functions as an inhibitor of the VieSA TCS. To test this we examined the effect of VieB on phosphotransfer between GST-VieS-C (GST-tagged cytoplasmic portion of VieS) and VieA-His_6._ In vitro phosphotransfer between an MBP-VieS-C fusion protein and VieA-His_6_ was previously demonstrated [13]. Using purified GST-VieS-C, we found that autophosphorylation and phosphotransfer to VieA-His_6_ behaves as expected (Figure 1A). When a five-fold molar excess of VieB was added to the reaction, there was a complete loss of the production of phosphorylated VieA-His_6_ (Figure 1A). VieB does not become readily phosphorylated in this assay even though it harbors a Rec domain, suggesting VieB does not act as a phosphate sink or compete with VieA-His_6_ for phosphorylation. However, our data so far cannot rule out the possibility that VieB may harbor high phosphatase activity, which could result in no observable phosphorylated VieB if it is a phosphatase of itself. Together, these data show that VieB is an inhibitor of phosphotransfer between GST-VieS-C and VieA-His_6_.

To examine whether VieB functions enzymatically, we varied its molar ratio relative to GST-VieS-C and P-VieA-His_6_ and measured phosphorylation of each protein. As seen in Figure 1B, while VieA-His_6_ is readily phosphorylated in the absence of VieB, as the VieB concentration increases, there was a correlative decrease in the amount of P-VieA-His_6_. At equal molar amounts, VieB was able to reduce the amount of P-VieA-His_6_ by approximately 50% (Figure 1B, lane 4). When VieB is increased to twice the molar amount, there was further loss of P-VieA-His_6_. Total inhibition occurred only when VieB was in molar excess over GST-VieS-C and VieA-His_6_ (Figure 1B, lane 7). In contrast, the amount of phosphorylated GST-VieS-C was largely unchanged in the reactions except for a modest decrease at the highest concentration of VieB (Figure 1B, lane 7). Taken together, these data suggest that VieB inhibition of VieA phosphorylation is dose-dependent and is not enzymatic.

### VieB inhibits VieS autophosphorylation and phosphotransfer

**Figure 2:**
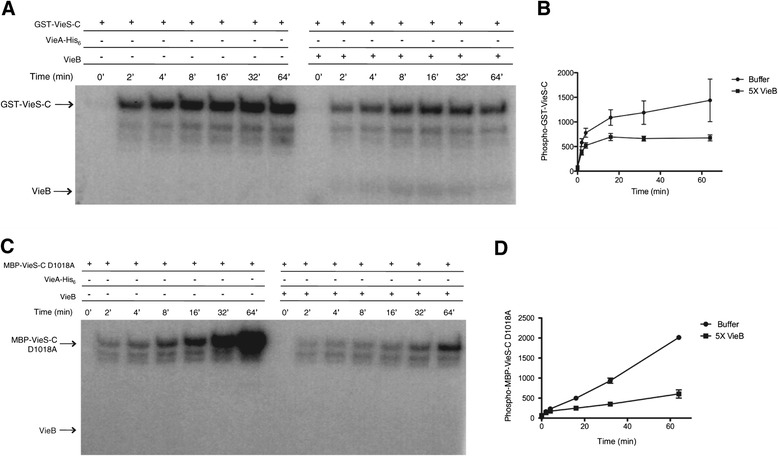
**VieB moderately inhibits VieS-C autophosphorylation.** Purified GST-VieS-C or MBP-VieS-C D1018A was incubated with ^32^P-ATP-γ in the presence or absence of 5 μM VieB over time at 30°C. Samples were stopped at indicated time points with the addition of 2X-denaturing sample buffer and separated using a 10% SDS-Page gel. GST-VieS-C labeled with ^32^P were observed by radioautography **(A,C)** and quantified in **(B,D)**.The radioautograph shown is a representative of three replicates. Error bars represent the SEM of three replicates.

To address the role of VieB in its ability to inhibit autophosphorylation of VieS, phosphotransfer experiments were conducted over time in the absence of VieA-His_6_. As seen in Figure 2A, wild-type GST-VieS-C was able to autophosphorylate in the presence of five-fold molar excess (5 μM) VieB, though the total amount of phosphorylation was lower (Figure 2A-B). These data could suggest that VieB is weakly affecting autophosphorylation. Alternatively, since VieS has three phosphorylation sites along its phosphorelay, it is also possible that VieB could block the intra-molecular phosphotransfer. If VieB blocks at the first or second site, the total amount of accumulated phosphate on VieS could decrease.

To test if VieB has inhibitory activity on the initial autophosphorylation of the HK domain, we used an intra-molecular phosphorelay mutant of VieS-C in the autophosphorylation assay. An MBP-VieS-C D1018A mutant was constructed that has the conserved Asp residue in the REC domain replaced with an Ala, rendering it only able to autophosphorylate at the HK domain while transfer between the REC-Hpt domains and the final transfer to VieA-His_6_ are disrupted. As seen in Figure 2C, while MBP-VieS-C D1018A was able to rapidly autophosphorylate to high levels over time in the absence of VieB; when VieB was present, the amount of autophosphorylation is significantly reduced (Figure 2C-D). Furthermore, the rate of autophosphorylation was lessened in the presence of VieB, confirming that VieB does inhibit VieS autophosphorylation, though it is not complete.

**Figure 3:**
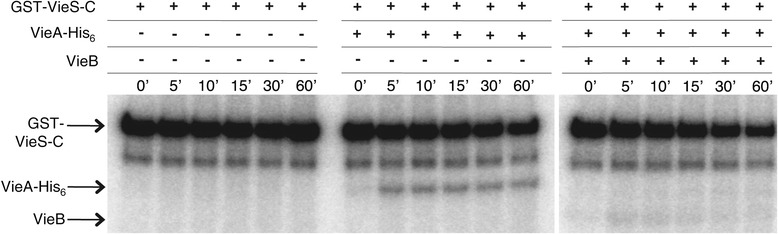
**VieB inhibits VieS intra-molecular phosphotransfer.** Purified GST-VieS-C was incubated with ^32^P-ATP-γ in the absence of VieA-His_6_ and VieB for 30 minutes at 30°C. Excess ^32^P-ATP-γ was removed from phosphorylated VieS-C constructs by gel filtration. P-VieS-C constructs were then incubated with additional MnCl_2_ and either buffer, 1 *μ*M VieA-His_6_ or pre-mixed 1 *μ*M VieA-His_6_ and 5 μM VieB for 60 minutes. Samples were stopped at indicated time points with the addition of 2X-denaturing sample buffer and separated using a 10% SDS-Page gel. VieS-C constructs labeled with ^32^P were observed by radioautography. The radioautograph shown is a representative of three replicates.

Since VieB is only able to partially inhibit GST-VieS-C autophosphorylation, yet can completely block phosphotransfer between VieS and VieA, we hypothesize that VieB must also be affecting either the VieS intra-molecular phosphorelay or phosphotransfer from the VieS Hpt domain to VieA. To test this hypothesis, the inhibitory effect of VieB on phosphotransfer between GST-VieS-C and VieA-His_6_ was observed in the absence of ATP. In order to specifically address phosphotransfer in the absence of autophosphorylation, ATP was removed from the reaction after GST-VieS-C was robustly phosphorylated to prevent further autophosphorylation. As seen in Figure 3, phosphorylated GST-VieS-C was able to transfer phosphate to VieA-His_6_ in the absence of ATP. However, when VieB was present, the amount of P-VieA-His_6_ was greatly reduced (Figure 3). This suggests that VieB is able to block phosphotransfer between VieS and VieA-His_6._ While we hypothesize that VieB may be inhibiting the intra-molecular phosphorelay between the VieS HK and REC domains, the data presented here cannot distinguish if VieB is acting intra- or inter-molecularly.

### VieB does not affect the stability of P-VieS-C

**Figure 4:**
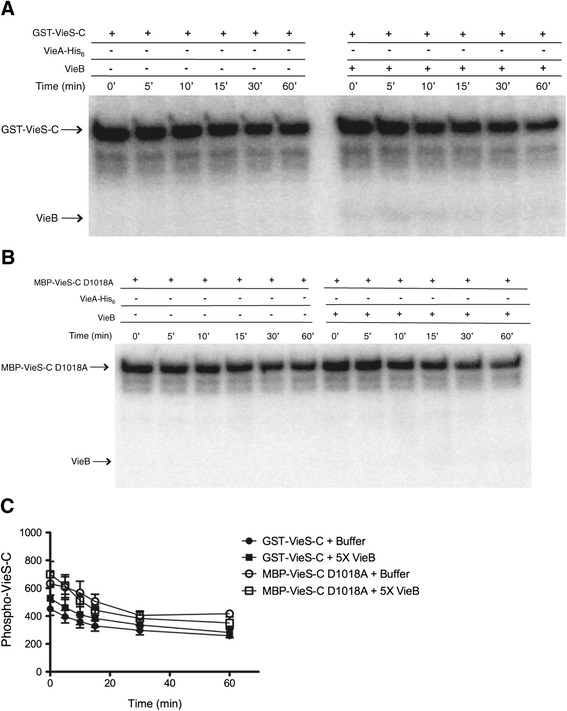
**VieB does not affect the stability of phosphorylated GST-VieS-C.** Purified GST-VieS-C or MBP-VieS-C D1018A was incubated with ^32^P-ATP-γ in the absence of VieB for 30 minutes at 30°C. Excess ^32^P-ATP-γ was removed from phosphorylated VieS-C constructs by gel filtration. P-VieS-C constructs were then incubated with additional MnCl_2_ and buffer or with 5 μM VieB for 60 minutes. Samples were stopped at indicated time points with the addition of 2X-denaturing sample buffer and separated using a 10% SDS-Page gel. VieS-C constructs labeled with ^32^P were observed by radioautography **(A, B)** and quantified in **(C)**.The radioautograph shown is a representative of three replicates. Error bars represent the SEM of three replicates.

Since VieB causes a reduction in the amount of P-VieS-C, VieB could affect the stability (auto-dephosphorylation) of P-VieS-C. To test this hypothesis, both wild-type GST-VieS-C and the mutant MBP-VieS-C D1018A were allowed to autophosphorylate in the absence of VieB and then the rate of loss of P-VieS-C was measured over time. As seen in Figure 4, the addition of VieB did not affect the loss of phosphate in either the wild-type GST-VieS-C or the MBP-VieS-C D1018A mutant. These data suggest that VieB does not alter the stability of phosphate on VieS-C, ruling out this mechanism of action for VieB.

### VieB is neither a phosphatase nor stimulates GST-VieS-C phosphatase activity

**Figure 5:**
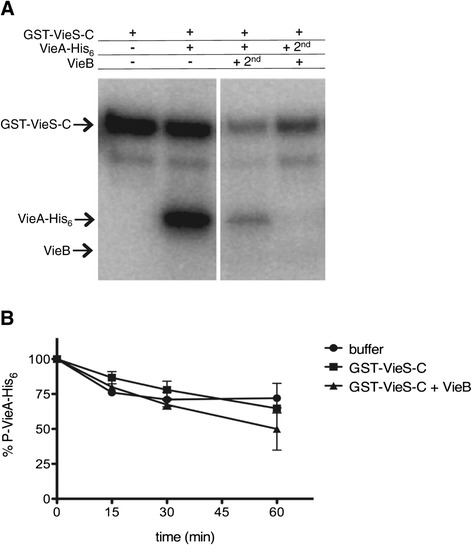
**VieB is not a phosphatase or stimulates GST-VieS-C phosphates activity. (A)** Previously described phosphotransfer assays were conducted at 30°C in the presence of ^32^P-ATP-γ with the following modifications: Lane 1- GST-VieS-C alone was incubated for 60 minutes, Lane 2- GST-VieS-C was incubated with 1 μM VieA-His_6_ for 60 minutes, Lane 3- GST-VieS-C was incubated with 1 μM VieA-His_6_ for 30 minutes then 5 μM wild-type VieB was added for an additional 30 minutes, Lane 4- GST-VieS-C was incubated with 5 μM wild-type VieB for 30 minutes then 1 μM VieA-His_6_ was added for an additional 30 minutes. Proteins labeled with ^32^P were observed by radioautography. The radioautographs shown are a representative of three replicates. **(B)** ^32^P-VieA-His_6_ was incubated with buffer, GST-VieS-C alone or GST-VieS-C and VieB for 60 minutes at 30°C. Samples were taken over time and quantified. 0 minute time point represents 100% ^32^P-VieA-His_6_. Error bars represent the SEM of three replicates.

Some auxiliary proteins that negatively modulate TCSs harbor phosphatase activity [22-24]. Since the addition of VieB has its most dramatic effect on the amount of P-VieA-His_6_, it is possible that VieB is a phosphatase of VieA-His_6_. To test this hypothesis, we conducted order of addition phosphotransfer experiments, where GST-VieS-C is incubated with either VieA-His_6_ or VieB prior to the addition of the other protein. When GST-VieS-C was first incubated with VieA-His_6_ and VieB was added second, P-VieA-His_6_ was still present, though to a much lesser degree than when VieB was absent (Figure 5A). However, some level of P-VieA-His_6_ remains even in the presence of VieB (Figure 5A). These data suggest that VieB is either inefficient in or not able to dephosphorylate P-VieA-His_6_. In contrast, when GST-VieS-C was incubated with VieB prior to the addition of VieA-His_6_, there was no detectable P-VieA-His_6_ suggesting a complete disruption of phosphotransfer (Figure 5A). Furthermore, GST-VieS-C was able to acquire phosphate even when VieB was present prior to VieA-His_6_. Taken together, these data suggest that VieB is not a phosphatase of VieA.

However, the observed reduction in phosphotransfer could be through the phosphatase activity of the SK. Therefore we next hypothesized that VieB binds GST-VieS-C and stimulates its phosphatase activity [25-27]. To test this hypothesis, we measured the rate of loss of radiolabeled P-VieA-His_6_ over time in the presence of GST-VieS-C, GST-VieS-C plus VieB, or in buffer alone. When P-VieA-His_6_ was incubated with buffer alone, there was an intrinsic slow rate of loss of phosphate to water (Figure 5B), which is a common phenomenon among RRs, though the rate of loss can vary [28,29]. When P-VieA-His_6_ was incubated with GST-VieS-C or with GST-VieS-C plus VieB, there was no difference in the rate of loss when compared to the buffer control (Figure 5B). Therefore these data suggest that GST-VieS-C does not have phosphatase activity nor does the presence of VieB stimulate that activity. It should be noted that in some instances, SK phosphatase activity requires the full-length protein folded properly in a membrane, therefore phosphatase activity of VieS in vivo cannot be completely ruled out by our data.

### Mutation of the conserved Asp residue in the REC domain affects the inhibitory activity of VieB

**Figure 6:**
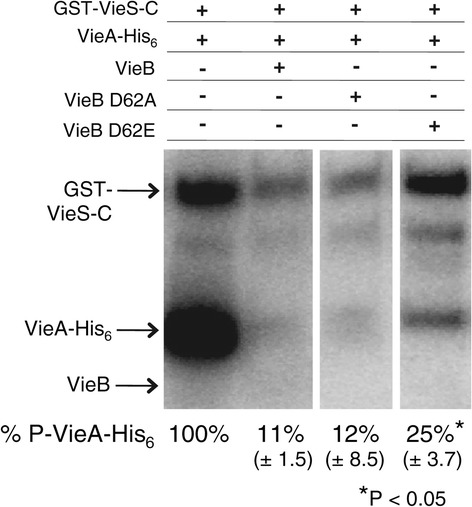
**Mutation of the conserved Asp residue affects the inhibitory activity of VieB.** GST-VieS-C was incubated with ^32^P-ATP-γ in the presence of equimolar VieA-His_6_ (lane 1) and either 5 μM wild-type VieB (lane 2), VieB D62A (lane 3), or VieB D62E (lane 4) for 30 minutes at 30°C. Samples were stopped with the addition of 2X-denaturing sample buffer and separated using a 10% SDS-Page gel. Proteins labeled with ^32^P were observed by radioautography. Figure panels are from the same experiment and exposed for the same amount of time. The radioautographs shown are a representative of four replicates. Standard deviation is shown below. Stars denotes a significant P-value < 0.05 determine by a Mann–Whitney U test.

In TCSs, the activity of the RR is regulated though the phosphorylation of a conserved Asp residue in the REC domain, resulting in activation of the RR and execution of its output activity. Due to the high sequence homology of the VieB REC domain to other REC domains and the presence of the conserved Asp (D62) residue, we hypothesized that phosphorylation of VieB affects its inhibitory activity. However, VieB was poorly phosphorylated by GST-VieS-C (Figure 1) or acetyl-phosphate (data not shown); our inability to achieve phosphorylated VieB presents a challenge for addressing if the inhibitory activity of VieB is regulated by its phosphorylation state. Therefore, as substitutes for unphosphorylated and phosphorylated VieB, point mutations in *vieB* were constructed to replace D62 with either an Ala (D62A), mimicking a non-phosphorylated state, or a Glu (D62E), which mimics a phosphorylated state. Of note, while the amount of wild-type VieB phosphorylation can vary, neither point mutant ever became phosphorylated during the phosphotransfer reactions, suggesting that the D62 residue is indeed the conserved site for phosphorylation and that there is no alternative phosphorylation site in VieB (Figure 6). When the purified VieB D62A was added to the reaction, this mutant was able to inhibit phosphotransfer to the same degree as wild-type VieB. However, the VieB D62E point mutant was a weaker inhibitor, allowing some amount of phosphotransfer to occur between GST-VieS-C and VieA-His_6_ (Figure 6). These data suggest that the phosphorylation state of VieB affects its ability to inhibit phosphotransfer, where unphosphorylated VieB is an active inhibitor while phosphorylated VieB is less active.

### VieB interacts with GST-VieS-C

Since VieB prevents phosphorylation of VieA-His_6_ through the inhibition of GST-VieS-C autophosphorylation and intra-molecular transfer, we hypothesize that VieB binds to GST-VieS-C. To address if VieB can interact with GST-VieS-C, GST pull-downs were conducted. Immobilized GST-VieS-C was able to pull down VieB and VieA-His_6_ from whole cell lysate (data not shown). To characterize this interaction, we used size-exclusion chromatography coupled with multi-angle light scattering (SEC-MALS) and composition-gradient MALS (CG-MALS) to quantify the self- and hetero-association affinity and stoichiometry for all combinations of the VieSAB proteins. Table 1 summarizes the results of these experiments. As revealed by the MALS data, VieS-C and VieA-His_6_ are both putative homo-dimers, and each VieS-C dimer binds a single VieA-His_6_ dimer. The equilibrium dissociation constant (*K*_*D*_) for this interaction is 1.38 μM. Additionally, VieB exists as a monomer, and each VieS-C dimer binds a single VieB monomer, with *K*_*D*_ = 0.467 μM. This affinity is approximately three-fold stronger than that of the VieS-C/VieA-His_6_ interaction, which is consistent with VieB being able to inhibit the VieS-VieA phosphotransfer. Of note, there were no other stoichiometries present for the VieB/VieS-C interaction, suggesting that only one VieB binding site is present on VieS-C. Similar to wild-type VieB, the VieB D62E point mutant is also a monomer. Unexpectedly, the VieB D62E, which is a weaker inhibitor of phosphotransfer, is still able to bind VieS-C at the same stoichiometry as wild-type VieB with a *K*_*D*_ of 0.197 μM. This affinity is about two-fold stronger than that of wild-type VieB. There is no detectable interaction between VieA-His_6_ and VieB. These data further support the model that VieB specifically interacts with VieS to inhibit phosphotransfer.

**Table 1:** **VieB specifically interacts with VieS-C**

**Protein Combination**	**Native Oligomeric State**		**Hetero-association stoichiometry**	***K***_**D**_ **(μM)**
VieS-C + VieA-His_6_	**VieS-C**	**VieA-His**_**6**_	1 dimer : 1 dimer	1.38 ± 0.35
	Dimer (MW = 150 kDa)	Dimer (MW = 130 KDa)		
VieS-C + VieB	**VieS-C**	**VieB**	1 dimer : 1 monomer	0.467 ± 0.054
	Dimer (MW = 150 kDa)	Monomer (MW = 64 kDa)		
VieS-C + VieB D62E	**VieS-C**	**VieB D62E**	1 dimer : 1 monomer	0.197 ± 0.061
	Dimer (MW = 150 kDa)	Monomer (MW = 64 kDa)		
VieA-His_6_ + VieB	**VieA-His**_**6**_	**VieB**	N/A	N/A
	Dimer (MW = 130 kDa)	Monomer (MW = 64 kDa)		

Characterization of VieS-C, VieA-His_6,_ wild-type VieB and the VieB D62E point mutant (self-association) were determined by Size-Exclusion Chromatography Multi-angle Light Scattering. To determine the protein-protein interactions of various VieSAB protein combinations, hetero-association interaction kinetics were determined over a range of protein concentrations by Composition-gradient Multi-angle Light Scattering. Data for the hetero-association stoichiometry are represented in monomer units. These data represent the average and ± SD of three independently purified replicates.

### VieB is a noncompetitive inhibitor

**Figure 7:**
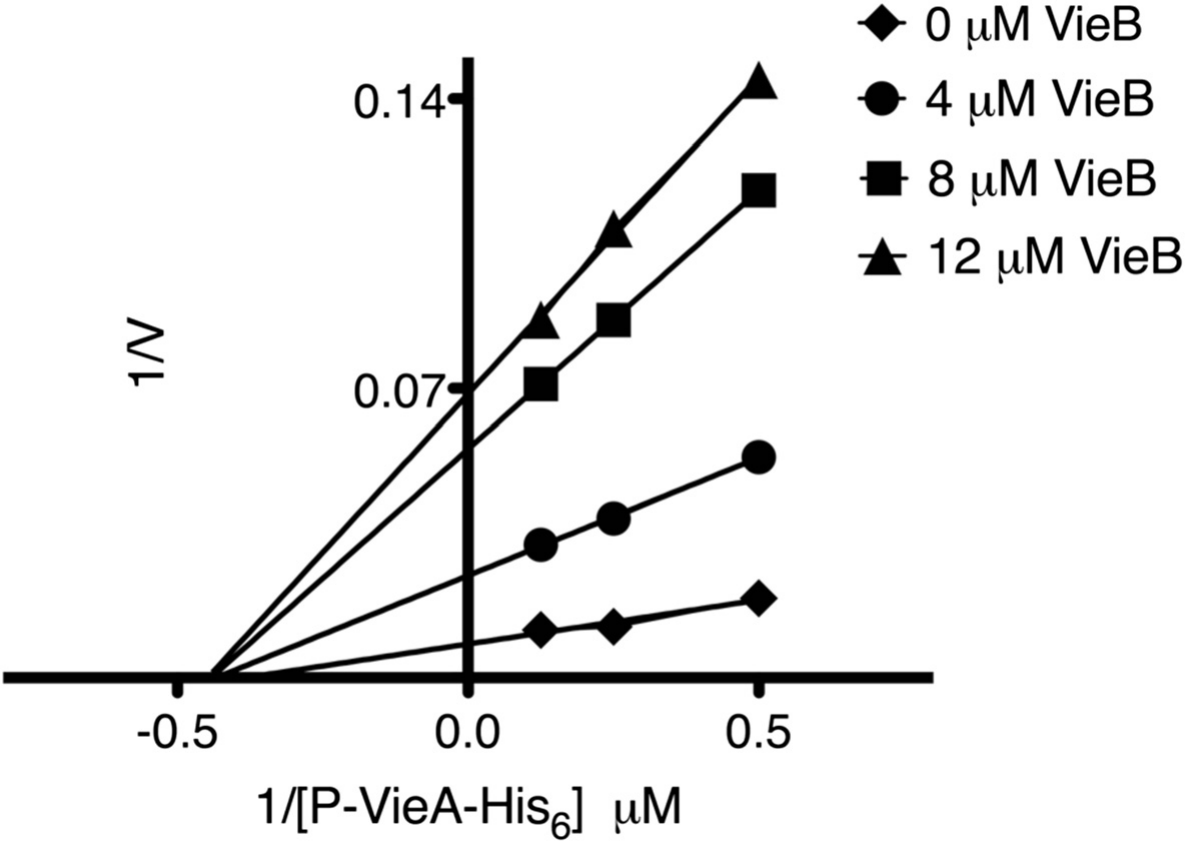
**VieB is a non-competitive inhibitor.** A Lineweaver-Burk Plot was generated using the previously described phosphotransfer assay over a range of VieA-His_6_ (2, 4, and 8 μM) and wild-type VieB (0, 4, 8, and 12 μM) concentrations. VieA-His_6_ phosphorylated with ^32^P (P-VieA-His_6_) was quantified by radioautography. These data represent the average velocity of three replicates (0, 4 and 12 μM VieB) and four replicates (8 μM VieB). V = velocity.

Based on the data presented so far, we propose that VieB functions as a phosphotransfer inhibitor by preventing GST-VieS-C autophosphorylation and phosphotransfer, however the mechanism of inhibition is unclear. Inhibitors are able to block enzymatic activity through two main mechanisms, competitive or noncompetitive binding. Previously, MBP-VieS-C was shown to have kinase activity, operating under second order Michaelis-Menten kinetics [13]. Therefore, to determine the mechanism of inhibition by VieB, we generated a Lineweaver-Burk Plot using phosphotransfer assays. VieB concentrations were varied to determine the rate (velocity) of phosphotransfer between GST-VieS-C and VieA-His_6_. Since VieB is a dose-dependent inhibitor, we expect that GST-VieS-C/VieA-His_6_ phosphotransfer will decrease as VieB concentrations increase. As expected, as the VieB concentration increases, the velocity at which GST-VieS-C can phosphorylate VieA-His_6_ decreases. Extrapolation of the line for each VieB concentration shows that all VieB concentrations result in the same Michaelis *K*_m_ (*x*-intercept) but varying *V*_max_ (*y*-intercept) (Figure 7). These data suggest that VieB is a noncompetitive inhibitor of GST-VieS-C.

### VieB does not affect the interaction between GST-VieS-C and VieA-His_6_

**Figure 8:**
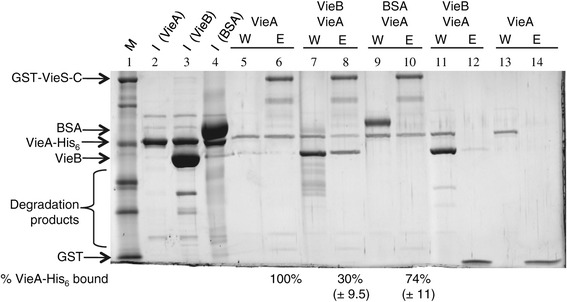
**VieB does not disrupt the GST-VieS-C/VieA-His**_**6**_ **interaction.** GST-VieS-C (lane 5–10) or GST alone (lane 11–14) bound to glutathione beads were incubated with either, pre-mixed 5 μM VieA-His_6_ and 25 μM VieB (VieB), 5 μM VieA-His_6_ alone (VieA) or 5 μM VieA-His_6_ and 25 μM BSA (BSA). Reactions were washed with 150 μl of wash buffer five times and protein was eluted off the beads with 150 μl of elution buffer containing reduced glutathione. Samples were taken from the input (I), wash (W) and elution (E) fractions for analysis on 10% SDS Page gel stained with Lumitein protein stain^TM^. VieB, VieA and BSA inputs are indicated by lanes 2–4, respectively. Percentage of VieA-His_6_ bound is the average of four replicates with standard deviation shown below; gel shown is a representative of the replicates. Statistical significance was determined by one-way ANOVA and Dunn’s Multiple Comparison. M = protein standard.

Given the noncompetitive model of inhibition, we would expect the binding of VieB to GST-VieS-C to have little to no effect on the binding ability of VieA-His_6_ to GST-VieS-C. To test this hypothesis, GST pull-down experiments with purified proteins were conducted. Since VieB was most effective at molar concentrations that are higher than GST-VieS-C and VieA-His_6_, we chose to conduct these experiments with VieB at a five-fold molar excess over VieA-His_6_, and with both VieA-His_6_ and VieB at molar excess over GST-VieS-C. When VieA-His_6_ was incubated with GST-VieS-C, VieA-His_6_ was pulled down in the elution fraction with GST-VieS-C (Figure 8, lane 6). Since VieA-His_6_ was in five-fold molar excess to GST-VieS-C, it was expected that some VieA-His_6_ would be present in the wash fraction (Figure 8, lane 5). The amount of VieA-His_6_ that was pulled down in this elution fraction was quantified and set to 100%. In the presence of VieB, the amount of VieA-His_6_ that was eluted with GST-VieS-C was significantly reduced by 70% (Figure 8, lane 8). However, BSA was used as a negative control to test if the addition of a nonspecific protein at the same five-fold excess molar concentration can prevent VieA-His_6_ binding. While there was a smaller reduction (26%) in the amount of VieA-His_6_ bound in the presence of BSA, this decrease is not significantly different from the decrease seen when VieB is present (as determine by one-way ANOVA and Dunn’s Multiple Comparison), suggesting that the reduction of VieA-His_6_ in both reactions are the result of nonspecific interactions. As a negative control for VieA-His_6_ and VieB nonspecific interactions with GST, GST alone was immobilized to the glutathione beads. As seen in Figure 8, lanes 11–14, neither VieA-His_6_ nor VieB interact with GST. Taken together, these data support the noncompetitive inhibition model, whereby the presence of VieB does not affect the ability of VieA-His_6_ to bind to GST-VieS-C but prevents only phosphotransfer.

## Discussion

VieSAB plays an important regulatory role in allowing *V. cholerae* to adapt to its environment as this pathogen transitions from the aquatic environment to the small intestine of the human host. VieSA composes a classical TCS, where VieS is a complex SK capable of autophosphorylating and engages in phosphotransferwith the RR, VieA [13]. In this study, we provide biochemical evidence that VieB, the previously uncharacterized third component in the VieSAB signal transduction system, inhibits phosphotransfer to VieA, thus providing a negative feedback mechanism to down-regulate the VieSA TCS phosphorelay. We show that VieB accomplishes this inhibition by binding to VieS and preventing autophosphorylation and phosphotransfer to VieA. Additionally, we show that mutating the conserved phosphorylation site in the REC domain can modulate the inhibitory activity of VieB, where the D62E mutant is a weaker inhibitor than the wild-type VieB or the D62A mutant.

**Figure 9:**
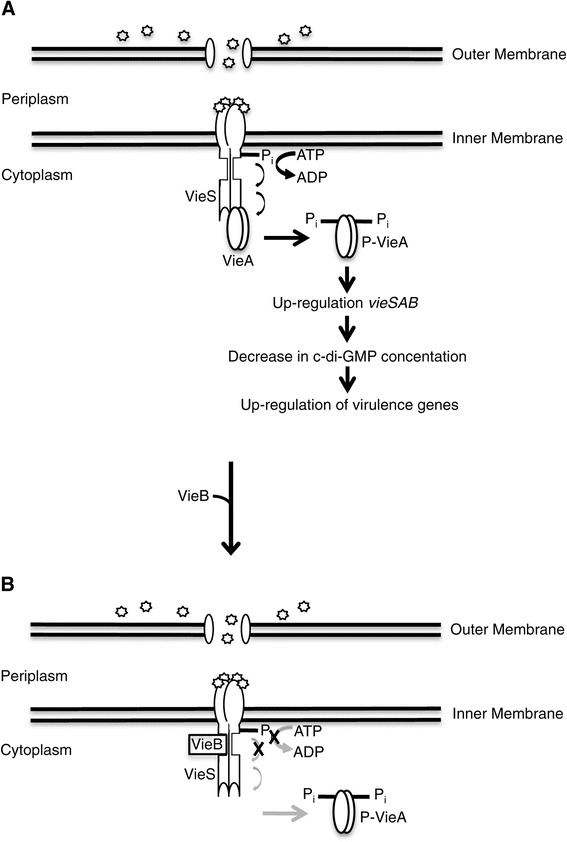
**Proposed model for VieB inhibition.** Stimulation of the VieSA TCS by binding of external signal (stars) to VieS results in autophosphorylation and phosphotransfer to and activation of VieA. We propose that VieA activation results in the amplification of the *vieSAB* operon and up-regulation of virulence genes. We hypothesize that this up-regulation of *vieA*, leads to decreased intracellular levels of c-di-GMP and enhanced expression of virulence genes **(A)**. Over time, or at high levels of transcription of the *vieSAB* operon, VieB accumulates. This pool of VieB is able to tightly bind, noncompetitively, to VieS. We hypothesize that this binding disrupts autophosphorylation and the transfer of phosphate between the HK and REC domains of VieS, down-modulating the phosphorelay. This lack of phosphotransfer to VieA results in down regulation of *vieSAB* and virulence genes **(B)**. ‘X’ denotes the inhibitory action of VieB. Black arrows correspond to active phosphotransfer while grey arrows denote incompletion of transfer.

*vieSAB* is well-described as an important contributor to *V. cholerae’s* control of gene expression during infection. The ability of VieSAB to modulate virulence gene expression is achieved through the VieA PDE domain, which hydrolyzes the secondary messenger molecule, cdGMP, during infection [14,15,20]. Changes in intracellular cdGMP are known to regulate gene expression, where low concentrations of cdGMP result in induction of virulence genes and repression of biofilm genes. Conversely, when cdGMP levels are high, the inverse is observed for these two classes of genes. VieA also contains a putative HTH DNA-binding domain, suggesting that in addition to its PDE activity, VieA may regulate gene expression through direct DNA binding, though this has not been shown. A previous microarray study suggests that VieA is autoregulatory, as a deletion of *vieA* resulted in decreased expression of the entire *vieSAB* operon when compared to wild type *V. cholerae* [30]. Taken together with this study, we present a working model incorporating the role of VieB in negatively regulating the expression of VieA through VieS inhibition (Figure 9). Upon VieS binding to an unknown environmental stimulus, VieS autophosphorylates at the HK domain, transfers the phosphate intra-molecularly, in order to phosphorylate the REC domain of VieA. Given VieA’s suggested autoregulatory activity, we propose that phosphorylation of VieA results in its activation as a transcription factor, triggering an increase in *vieSAB* expression. This up-regulation increases the quantity of VieA in the cytoplasm resulting in reduction of cdGMP concentration through enzymatic hydrolysis by the VieA PDE domain. This decrease in the intracellular level of cdGMP helps trigger expression of virulence genes needed for survival in the host (Figure 9A).

Over time, autoregulation of the *vieSAB* operon results in accumulation of VieB. We hypothesize that VieB is able to tightly bind VieS as a noncompetitive inhibitor, ultimately preventing additional phosphotransfer to VieA. We propose that this disruption of VieA phosphorylation is due to VieB binding at or near the HK domain of VieS and inhibiting both its autophosphorylation and intra-molecular transfer of phosphate to its REC domain. Phospho-VieA levels are then presumably reduced, shutting off or at least down-regulating expression of the *vieSAB* operon and virulence genes due to increasing cdGMP levels (Figure 9B). Since VieB appears to be a moderate inhibitor of autophosphorylation, it is possible for some VieS to acquire phosphate at the HK domain, even in the presence of VieB. Therefore, if VieS becomes phosphorylated, the ability of VieB to not only prevent further autophosphorylation but also disrupt the transfer of the already bound phosphate ensures that VieA does not become phosphorylated.

Negative feedback regulation is not uncommon among TCSs. Indeed, other TCS have incorporated auxiliary proteins in order to inhibit the phosphosignalling between the SK and RR. For example, in *B. subtilis*, numerous auxiliary proteins negatively control the progression of phosphotransfer in a TCS that controls sporulation [22-24]. The inhibitors Sda and KipI interact with the SK, KinA, binding to the HK domain to block autophosphorylation, specifically the transfer of phosphate from ATP to the His residue. However, neither block phosphotransfer to the RR nor dephosphorylate the SK [23,31]. In addition to Sda and KipI, Rap proteins and Spo0E act as phosphatases at various stages in the phosphotransfer and dephosphorylate two important RRs, Spo0F and Spo0A. Another example of inhibition by auxiliary proteins is modulation of the SK, NtrB, by the PII nitrogen-sensor protein in the NtrBC TCS in *E. coli* [32]. NtrBC is involved in the assimilation of ammonia and metabolism of alternative nitrogen-containing compounds. NtrB has dual functions, harboring both kinase and phosphatase activities, where the directional flow of phosphate depend on its interaction with PII. When PII is bound to NtrB, this promotes phosphatase activity on the RR, NtrC. Conversely, when PII is not interacting with NtrB, this allows NtrC to act as a kinase to phosphorylate NtrC [33]. We show that VieB interacts specifically with the SK, VieS, however our data suggest that VieB is neither a phosphatase nor does it modulate the phosphatase activity of VieS. While the function of VieB appears similar to the Sda and KipI inhibitors in *B. subtilis*, our data provide evidence that is also capable of blocking either the intra-molecular phosphorelay or phosphotransfer to VieA. Therefore, we believe that the ability of VieB to specifically block both autophosphorylation and phosphotransfer provides a novel mechanism for the negative regulation of TCS.

Our data suggests that the inhibitory activity of VieB may be regulated, which to our knowledge is a novel mechanism for regulating an auxiliary inhibitor of a TCS. Just as the activity of traditional RRs is controlled by the phosphorylation of the REC domain, it appears that VieB also may be controlled by this same mechanism. However, for traditional RRs, phosphorylation leads to activation, whereas for VieB, phosphorylation (mimicked by the D62E mutation) leads to inactivation. This reverse regulation by phosphorylation is an attractive model for an inhibitor like VieB: Since VieB in its unphosphorylated state is able to bind VieS and is active for inhibition, phosphorylation of VieB can then serve as a shut-off signal, allowing VieS to regain function. Intriguingly, the D62E mutant retains strong binding to VieS. This suggests that the mere binding of VieB to VieS is not sufficient for inhibition, but that the structure of VieB that is bound plays an important role. However, we cannot rule out the possibility that the D62E mutant fails to mimic the phosphorylated form of VieB adequately.

## Conclusions

Our work presented here biochemically characterizes the role of VieB in the VieSA TCS. We show that VieB binds to the VieS sensor kinase and blocks phosphotransfer to the VieA response regulator and cdiGMP phosphodiesterase. Thus, VieB provides what we believe is a novel negative feedback mechanism for controlling a TCS, allowing for tight regulation over VieA activity and subsequent cdGMP levels. This further highlights the role of this TCS in modulating gene expression in *V. cholerae* and provides a more comprehensive understanding of how VieSAB functions. Future work is necessary for elucidating structural details of the interaction between VieB and VieS, as well as the precise conditions under which VieB is active. Insight into how VieSAB modulates the inverse expression of virulence and environmental genes may aid in the understanding of how and when *V. cholerae* regulates its complex virulence cascade as it transitions from the aquatic environment into the host.

## Methods

### Growth conditions and strain construction

All strains used in this study are listed in Table 2. All PCR primers used are listed in Table 3. Bacteria were grown in Luria-Bertani broth supplemented with 100 μg/ml ampicillin at 37°C with aeration. 1 mM isopropyl-β-D-thiogalactopyranoside (IPTG) was added when necessary to induce protein expression. The cytoplasmic encoding portion of *vieS* (*vieS-C*) and full-length *vieB* (*vieB*) were constructed using PCR amplicons made from *V. cholerae* strain O395 template DNA. For glutathione *S*-transferase (GST) tagged *vieS-C*, a silent mutation was created at the internal NdeI restriction endonuclease recognition site at position 2916. This mutant allele was generated by overlap extension PCR [34] using primer pairs GST-VieS-C F/GST-VieS-C T2916C R and GST-VieS-C T2916C F/GST-VieS-C R. *vieB* was generated by PCR using the VieB F/R primers. The outer primers for each of these alleles introduce NdeI and BamHI restriction sites for subsequent cloning. After amplification, PCR products were double-digested with NdeI and BamHI (New England Biolabs). Generation of the GST-*vieS-C* was ligated into a modified pGEX vector that includes a TEV protease recognition site after the GST tag [6]. *vieB* was ligated into a modified pET15b plasmid that also includes a TEV recognition site after the poly-Histidine (His_6_) tag [6]. The maltose binding protein (MBP) tagged *vieS-C* D1018A mutant was generated using overlap extension PCR using the primer pairs MBP-VieS-C F/MBP-VieS-C D1018A R and MBP-VieS-C D018A F/MBP-VieS-C R. Specifically for MBP-*vieS-C* D1018A, the outer primers introduced a BamHI and SalI restriction sites for subsequent cloning. Generation of the MBP-*vieS-C* D1018A was ligated into the pMALc2E vector. Ligations were transformed into *E. coli* DH5α by electroporation and plated on Luria-Bertani broth supplemented with ampicillin. The insert in each plasmid was confirmed by DNA sequencing. The pMMB67EH::*vieA-His*_*6*_ was purified from DH5α and transformed into *E. coli* BL21 (DE3) for protein expression [35]. The VieB D62 point mutants were constructed using the QuickChange site-directed mutagenesis method (Stratagene) using the pET15b::His_6_-*vieB* vector. The mutations were confirmed by DNA sequencing, and the plasmids were transformed into *E. coli* BL21 (DE3) for protein expression.

**Table 2:** **Bacterial strains and plasmids used in this study**

**Strain**	**Description**	**Protein expressed**	**Reference/Source**
AC50	*V. cholerae* classical O395 biotype	N/A	4
AC4713	*E. coli* BL21 (DE3) pGEX-TEV::*vieS-C*	GST-VieS-C	This study
AC4714	*E. coli* BL21 (DE3) pMMB67eh::*vieA-His*_*6*_	VieA-His_6_	32
AC4715	*E. coli* BL21 (DE3) pET15b-TEV::His_6_-*vieB*	His_6_-VieB	This study
AC4716	*E. coli* BL21 (DE3) pET15b-TEV::His_6_-*vieB D62A*	His_6_-VieB D62A	This study
AC4717	*E. coli* BL21 (DE3) pET15b-TEV::His_6_-*vieB D62E*	His_6_-VieB D62E	This study
AC4002	*E. coli* BL21 (DE3) pMalc2E-TEV::*vieS-C*	MBP-VieS D1018A	13

**Table 3:** **Primers used in this study**

**Primer Name**	**Primer Sequence 5′ − 3′**
GST-VieS-C F	tcactgtg*catatg*actgagcagctacgttggttgacgg
GST-VieS-C R	gagcgagtc*ggatcc*tcagagataacgactgagtactttgcgc
GST-VieS-C T2916C F	gttgattactgactgccacatgccacatcttgatg
GST-VieS-C T2916C R	catcaagatgtggcatgtggcagtcagtaatcaac
VieB F	tcactgtg*catatg*gctgtacctacttttgctgaattaaaag
VieB R	gagcgagtc*ggatcc*ttacgcctcaactgattcgcttcgc
VieB D62A F	gatttgatatttttatttgcgcttacaacttcggtaaggggtt
VieB D62A R	aaccccttaccgaagttgtaagcgcaaataaaaatatcaaatc
VieB D62E F	gatttgatatttttatttgcgagtacaacttcggtaaggggtt
VieB D62E R	aaccccttaccgaagttgtactcgcaaataaaaatatcaaatc
MBP-VieS-C F	CGC*GGATCC*TTACGCAGCTCCGAACAAG
MBP-VieS-C R	ACGC*GTCGAC*TTATTCGCTCTGATACTGATG
MBP-VieS-C D1018A F	TATGGCAATTGGTAATCAACAAATCATACTGCTCAGGATGTTGCGAGAGCTTTT
MBP-VieS-C D1018A R	CGAAAAGCTCTCGCAACATCCTGAGCAGTATGATTTGTTGATTACCAATTGCCA

All primers are listed 5′ to 3′. Italics indicate restriction enzymes used for cloning.

### Protein expression and purification

*E. coli* BL21 (DE3) containing the protein expression vectors were grown in 1 L cultures to an OD_600_ = 0.5-0.8. Protein expression was induced by the addition of 1 mM IPTG and grown at 20°C for 17 hours. 1 L cell cultures were harvested by centrifugation at 2, 990 × *g* for 20 minutes and resuspended in 25 ml of the following buffers: for GST-VieS-C and MBP-VieS-C D1018A, 20 mM Tris pH = 8, 150 mM NaCl, 5 mM *beta* mercaptoethanol (βME), Complete EDTA-free protease inhibitor cocktail tablet (Roche); and for VieA-His_6_, His_6_-VieB, and His_6_-VieB D62 mutants, 20 mM Tris pH = 8, 150 mM NaCl, 25 mM Imidazole, 5 mM βME, Complete EDTA-free protease inhibitor cocktail tablet (Roche). The resuspended cells were lysed on ice with five, 30-second pulses (0.5 seconds on, 0.5 second off) of sonication at 50% amplitude with one-minute rest in between pulses. Cell debris was pelleted by centrifugation at 38, 464 × *g* for 45–60 minutes at 4°C and the supernatant was collected.

For GST-VieS-C, supernatant was incubated on 7 ml of Glutathione Sepharose 4B beads (GE Healthcare) for 30 minutes at 4°C. The beads were washed with three column volumes of 20 mM Tris pH = 8, 200 mM NaCl, 5 mM βME. Protein was eluted in 30 ml of 100 mM Tris pH = 8, 20 mM reduced glutathione, 100 mM NaCl, 5 mM βME. For VieS-C, the GST tag was removed by the addition of TEV protease overnight at 4°C. Both GST-VieS-C and VieS-C were diluted three-fold with Buffer A (20 mM Tris pH = 8, 1 mM DTT) and applied directly to a 4 ml Source15Q anion exchange column (GE Healthcare) that has been equilibrated in Buffer A. Protein was eluted using a 0-50% (v/v) Buffer B (20 mM Tris pH = 8, 1 M NaCl, 1 mM DTT) gradient developed over 15 column volumes, with protein eluting around 33% (v/v). The peak fractions were pooled and concentrated by centrifugation at 2,514 × *g* at 4°C using the Amicon Ultra-15 10 K centrifugal filters (Millipore). Protein was then applied to a Superose12 prep grade 16/70 gel filtration (GE Healthcare) column that was equilibrated in Gel Filtration Buffer (25 mM Tris pH = 7.5, 100 mM KCl, 1 mM DTT, 5% glycerol (v/v)). For VieS-C, peak fractions were pooled and incubated a second time on 2 ml of Glutathione Sepharose 4B beads equilibrated in Gel Filtration Buffer for 30 minutes at 4°C to remove any remaining GST tag or GST-VieS-C protein. Flow-through containing VieS-C was collected and concentrated.

For MBP-VieS-C D1018A, supernatant was incubated on 5 ml of amylose high flow resin (New England Biolabs) for 30 minutes at 4°C. Beads were washed with five column volumes of 20 mM Tris pH = 8, 150 mM NaCl, 5 mM βME and eluted in 30 ml of 20 mM Tris pH = 8, 100 mM NaCl, 1 mM DTT and 30 mM maltose. Protein was diluted three-fold with Buffer A and applied to a 4 ml Source15Q anion exchange column. MBP-VieS-C D1018A was eluted using a 0-50% (v/v) Buffer B gradient developed over 15 column volumes, eluting around 31% (v/v). The peak fractions were pooled, concentrated and applied to a Superose12 prep grade 16/70 gel filtration column equilibrated in Gel Filtration Buffer.

For VieA-His_6_, His_6_-VieB and His_6_-VieB D62 point mutants, supernatant was incubated on 7 ml of His-Pur NiNTA beads (Thermo Scientific) for 30 minutes at 4°C. Beads were washed three times with three column volumes of Wash 1 (20 mM Tris8, 150 mM NaCl, 25 mM imidazole, 5 mM βME), three times with three column volumes of Wash 2 (same as Wash 1 except 50 mM imidazole), and eluted in 30 ml of Elution Buffer (same as Wash 1 except 300 mM imidazole). For VieB and the D62 point mutants, the His tag was removed by the addition of TEV protease overnight at 4°C. Proteins were diluted three-fold with Buffer A and applied to a 4 ml Source15Q anion exchange column. VieA-His_6_ was eluted using a 0-40% (v/v) Buffer B gradient (eluting around 23% (v/v)) while VieB and the D62 mutants were eluted using a 0-35% (v/v) Buffer B gradient (eluting around 20% (v/v)), both developed over 15 column volumes. The peak fractions were pooled, concentrated, and applied to a Superose12 prep grade 16/70 gel filtration column equilibrated in Gel Filtration Buffer.

### Phosphotransfer assays

Phosphotransfer reactions were carried out as described by Martinez et al. [13] with the following modifications. Phosphotransfer reactions were incubated in phosphotransfer buffer (Gel Filtration Buffer supplemented with 5 mM MnCl_2_, 25 μM radiolabeled ^32^P-ATP-γ [10 mCi/ml; Perkin Elmer, Boston, MA] and 1.25 μM cold ATP). GST-VieS-C, MBP-VieS-C D1018A and VieA-His_6_ were at final concentrations of 1 μM while VieB and the D62 mutants were at a final concentration of 5 μM, unless stated otherwise. Once reactions were stopped by the addition of 2X denaturing sample buffer (10 mM Tris pH = 6.8, 4% (w/v) SDS, 20% (v/v) glycerol, bromophenol blue, βME), proteins were separated on 10% SDS-PAGE gel at 200 V for 45 minutes. Time course experiments were stopped at indicated time points shown. Autoradiographs were recorded and quantified with FUJI FILM Image Reader FLA-9000 (Life Science) and FUJI FILM Multi-Gauge software (Life Science). Due to inherent experimental variation, an internal control of GST-VieS-C and VieA-His_6_ phosphotransfer in the absence of VieB is included with every experiment.

### Phosphorylated VieS stability assay

1 μM GST-VieS-C or MBP-VieS-C D1018A was incubated with 25 μM ^32^P-ATP-γ for 30 minutes at 30°C. To remove excess ATP, phosphorylated VieS-C (P-VieS-C) constructs were passed through a Performa DTR spin gel filtration column (EdgeBio) for two minutes at 750 × *g*. Additional MnCl_2_ was added and Gel Filtration Buffer or 5 μM VieB was added to P-VieS-C constructs and incubated at 30°C. The amount of P-VieS-C was measured over time. At indicated time points, reactions were stopped and analyzed as previously described for the phosphotransfer assays.

### Phosphorylated VieS transfer assay

1 μM GST-VieS-C was incubated with 25 μM ^32^P-ATP-γ for 30 minutes at 30°C. To remove excess ATP, P-VieS-C constructs were passed through a Performa DTR spin gel filtration column (EdgeBio) for two minutes at 750 × *g*. Additional MnCl_2_ was added and then either Gel Filtration Buffer, 1 μM VieA-His_6_, 5 μM VieB or pre-mixed 1 μM VieA-His_6_ and 5 μM VieB was added to P-VieS-C and incubated at 30°C. The amount of P-VieS-C was measured over time. At indicated time points, reactions were stopped and analyzed as previously described for the phosphotransfer assays.

### VieS phosphatase assay

GST-VieS-C was bound to Glutathione Sepharose 4B beads equilibrated in phosphotransfer buffer. Beads were washed and VieA-His_6_ was added in the presence of 25 μM radiolabeled ^32^P-ATP-γ and incubated for 30 minutes at 30°C. Beads were washed three times with 150 μl of 25 mM Tris pH = 7.5, 150 mM KCl, 1 mM DTT by centrifugation for 30 seconds at 12, 000 × *g* using Pierce Spin Columns (Thermo Scientific) to collect phosphorylated VieA-His_6_ (P-VieA-His_6_). P-VieA-His_6_ was incubated with new GST-VieS-C alone or GST-VieS-C plus VieB in phosphotransfer buffer in the absence of [^32^P-γ]. Samples were collected over time and reactions were analyzed and quantified using the described method above for the phosphotransfer assays.

### Lineweaver-Burk plot

Phosphotransfer assays described above were used to generate the Lineweaver-Burk plot except VieA-His_6_ concentrations ranged from 2–8 μM and VieB concentrations ranged from 0–12 μM while GST-VieS-C remained constant (1 μM). Samples were taken at 0, 3.5, 7.5, and 15 minutes. The amount of phosphorylated VieA-His_6_ was quantified over time in order to generate enzyme reaction velocities at each concentration combination. A double reciprocal plot was generated by plotting the inverse of the enzyme reaction velocity against the inverse of VieA-His_6_ (substrate) concentration using GraphPad Prism5 (GraphPad). Extrapolation of each line to determine the intercept was determined by linear regression analysis.

### Multi-angle light scattering

Size-exclusion chromatography coupled with multi-angle light scattering (SEC-MALS) was used to determine oligomeric state of each protein. SEC-MALS was conducted using the ÄKTA HPLC Explorer system (GE Healthcare) connected to a vacuum degasser (Alltech), a Superose12 10/300 GL gel filtration (GE Healthcare) column equilibrated in 0.1 μm filtered Gel Filtration Buffer that is directly connected to a DAWN HELEOS II light scattering detector (Wyatt Technology Corporation) and an Optilab T-rEX refractive index detector (Wyatt Technology Corporation). The column temperature was held at 4°C while light scattering detection was conducted at 25°C. A MALS baseline was established with Gel Filtration Buffer overnight. Light scattering and concentration data were acquired and analyzed with ASTRA software (version 6, Wyatt Technology Corporation) to determine molar mass in solution, and thus oligomeric sate, of each protein. All light scattering experiments were conducted using the untagged VieS-C protein, which is able to dimerize in the absence of the GST tag.

Composition gradient MALS (CG-MALS) was conducted to probe the hetero-association interactions between various combinations of the VieSAB proteins [36-38]. The Calypso II syringe pump system (Wyatt Technology Corporation) was used to inject various protein concentration mixtures to the DAWN HELEOS II light scattering detector that was attached to the UV detector of the ÄKTA HPLC Explorer system (GE Healthcare). The Gel Filtration Buffer was used for all CG-MALS and was filtered through a 0.1 μm filter. Proteins were diluted into this Gel Filtration Buffer at a predetermined stock concentration and were subsequently filtered through a 0.02 μm filter. After each injection, the flow was stopped for 30–180 seconds to allow protein interactions to reach equilibrium. Hetero-association stoichiometry, and dissociation constants (*K*_*D*_) were determined using the CALYPSO software (version 2.1.3, Wyatt Technology Corporation) based off the light scattering and UV signal acquired during the stopped flow for each concentration gradient. All CG-MALS experiments were conducted using the same untagged VieS-C protein used in SEC-MALS.

### GST pull-down assays

Unconjugated GST tag, used as a negative control for purified protein GST pull-downs, was purified as follows: TEV protease was added to previously purified GST-VieS-C overnight at 4°C. The sample was incubated on 500 μl of Glutathione Sepharose 4B beads that were equilibrated in Gel Filtration Buffer for 30 minutes at 4°C. Beads were washed and eluted as described above for GST-VieS-C. Protein was directly applied to a HiLoad 16/60 Superdex75 prep grade gel filtration (GE Healthcare) column to separate GST alone from any un-cleaved GST-VieS-C protein. The peak fractions corresponding to GST tag alone were collected and used for subsequent experiments.

For pull-downs, 1 μM of either GST-VieS-C or GST tag was incubated on 50 μl Glutathione Sepharose 4B beads for 30 minutes at 4°C using Pierce Spin Columns. Bound protein was washed three times with 150 μl of 25 mM Tris pH = 7.5, 150 mM KCl, 1 mM DTT by centrifugation for 30 seconds at 12, 000 × *g*. 5 μM VieA-His_6_ that was premixed with either buffer, 25 μM VieB, or 25 μM BSA was incubated on the beads for 15 minutes at 30°C. Reactions were washed five times each with three column volumes of 25 μM Tris pH = 7.5, 100 mM KCl, 1 mM DTT using the same centrifugation conditions. An additional dry spin was conducted to ensure all of the wash was removed from the column. GST-VieS-C or GST and all bound proteins were eluted using 150 μl of the elution buffer described above for GST-VieS-C purification. Samples from the input, wash and elution fractions were taken, 2X denaturing sample buffer was added, and were analyzed by 10% SDS-PAGE gel that was stained with Lumitein protein stain™. Gel images were acquired using the FUJI FILM Image Reader FLA-9000 and protein bands were quantified using FUJI FILM Multi-Gauge software.

## Abbreviations

TCS: Two-component system
SK: Sensor kinase
RR: Response regulator
REC: Receiver
HTH: Helix-turn-helix
cdGMP: Cyclic-di-GMP
CT: Cholera toxin
PDE: Cyclic-di-GMP phosphodiesterase
GST: Glutathione S-transferase
IPTG: Isopropyl- isothiogalactopyranoside; iogalbeta mercaptoethanol
SEC-MALS: Size-exclusion chromatography - multi-angle light scattering
CGMALS: Composition gradient - multi-angle light scattering
His6: Poly-Histidine
